# Exposure to airborne cadmium and breast cancer stage, grade and histology at diagnosis: findings from the E3N cohort study

**DOI:** 10.1038/s41598-021-01243-0

**Published:** 2021-11-29

**Authors:** Amina Amadou, Delphine Praud, Thomas Coudon, Aurélie M. N. Danjou, Elodie Faure, Floriane Deygas, Lény Grassot, Karen Leffondré, Gianluca Severi, Pietro Salizzoni, Francesca Romana Mancini, Béatrice Fervers

**Affiliations:** 1 Département Prévention Cancer Environnement, Centre Léon Bérard, 28 rue Laënnec, 69373 Lyon Cedex 08, France; 2 Inserm U1296 Radiations: Défense, Santé, Environnement, Lyon, France; 3 Ecole Centrale de Lyon, INSA Lyon, Université Claude Bernard Lyon 1, Ecully, France; 4 Université de Bordeaux, ISPED, Inserm U1219, Bordeaux Population Health Center, Bordeaux, France; 5 Paris-Saclay University, UVSQ, Univ. Paris-Sud, Inserm, Gustave Roussy, Team “Exposome and Heredity”, CESP, 94805 Villejuif, France; 6 Departement of Statistics, Computer Science and Applications (DISIA), University of Florence, Florence, Italy

**Keywords:** Cancer, Environmental sciences, Risk factors

## Abstract

Molecular studies suggest that cadmium due to its estrogenic properties, might play a role in breast cancer (BC) progression. However epidemiological evidence is limited. This study explored the association between long-term exposure to airborne cadmium and risk of BC by stage, grade of differentiation, and histological types at diagnosis. A nested case–control study of 4401 cases and 4401 matched controls was conducted within the French E3N cohort. A Geographic Information System (GIS)-based metric demonstrated to reliably characterize long-term environmental exposures was employed to evaluate airborne exposure to cadmium. Multivariable adjusted odds ratios (OR) and 95% confidence intervals (CI) were estimated using conditional logistic regression models. There was no relationship between cadmium exposure and stage of BC. Also, no association between cadmium exposure and grade of differentiation of BC was observed. However, further analyses by histological type suggested a positive association between cadmium and risk of invasive tubular carcinoma (ITC) BC [OR_Q5 vs Q1_ = 3.4 (95% CI 1.1–10.7)]. The restricted cubic spline assessment suggested a dose–response relationship between cadmium and ITC BC subtype. Our results do not support the hypothesis that airborne cadmium exposure may play a role in advanced BC risk, but suggest that cadmium may be associated with an increased risk of ITC.

## Introduction

Worldwide, breast cancer (BC) is the most frequent cancer and the leading cause of cancer death among women worldwide, with an estimated 2.3 million new cases in 2020^1^. BC is a heterogeneous disease at the histopathological and molecular levels, comprising different subtypes defined by their distinct histological, biological features, and clinical behaviors^2,3^. Current evidence suggests etiological heterogeneity with differential effects of risk factors on hormone receptor status, pathological grade, stage or histological type at diagnosis^4,5^. For example, increased risks related to hormone and reproductive factors (including age at menarche, older age at first birth, endogenous estrogens) have been consistently found for Estrogen receptor positive (ER+) and/or Progesterone receptor positive (PR+) breast tumors^6,7^. In addition to well-known reproductive and lifestyle factors^8,9^, a growing number of studies has identified an increased risk of BC associated with exposure to environmental pollutants, in particular to pollutants with endocrine disrupting properties; and linked exposures to several endocrine-disrupting-chemicals (EDC) to tumors size, lymph nodes involvement, and development of tumor metastasis and other hallmarks of BC tumor aggressiveness^10–13^.

Molecular and cellular studies suggest that cadmium due to its estrogenic properties, might play an important role in BC progression, tumor growth and invasion, as well as enhances the migratory ability of metastatic cells^14–16^. Cadmium is an environmental contaminant that exerts toxic effects promoting several cancers including possibly BC^17,18^. It is classified as a group 1 human carcinogen by the international agency for research on cancer (IARC)^19^. Cadmium is emitted to the atmosphere from both natural and anthropogenic sources, including mainly tobacco smoking, mining, smelting and refining of non-ferrous metals, and waste incineration^18,20^. Major route of cadmium exposure involve inhalation or incidental ingestion from contaminated hands, food, or cigarettes^18,21^. Compared to dietary exposure, inhalation is a far smaller source of cadmium exposure except for smokers. However compared to ingested cadmium, a larger proportion of inhaled cadmium is retained by the body. Also, 2.0–6.0% of ingested cadmium is absorbed by the body while in the case of inhalation, 30.0–60.0% is absorbed^21^. Accordingly, airborne cadmium has been suggested to contribute substantially to overall cadmium exposure^21^.

Ponce et al. reported that chronic cadmium treatment increases cell spreading and cell migration and promotion^16^. Also, a positive association of blood cadmium levels with distant metastasis has been reported^22^. Other experimental studies reported that cadmium stimulated cell proliferation by activating genes and signals associated with cancer migration and invasion BC cells^23,24^. Wei et al. have shown that low concentrations of cadmium transformed the non-tumorigenic breast epithelial cells to a more mesenchymal-like morphology, suggesting a potential role of cadmium in promoting metastasis^25^. Moreover, in vitro studies suggested that cadmium might contribute to enhancing the aggressiveness of breast tumors through stimulation of the transcription of oncogenic c-Myc and downregulating the tumor suppressor p21^26^. Furthermore, a study investigating cadmium concentrations in BC tissues, reported that cadmium was positively associated with histological type of tumor, its size and grading^27^. In the study by Tang et al. the authors assessed the toxic effect of cadmium in a TNBC MDA-MB-231 cell exposure, and observed that cadmium inhibited the viability of MDA-MB-231 cells in a time- and dose-dependent manner, while gasdermin E -activated pyroptosis in cadmium toxicity, suggesting a potential impact in triple-negative BC^28^. However, current epidemiological studies have provided inconsistent evidence regarding the association of cadmium air pollution exposure with BC risk overall^29,30^ and no epidemiological study to date has investigated the impact of cadmium exposure on BC stage, grade of differentiation, or histological type at diagnosis. A recent dose–response meta-analysis of cohort studies of 10 studies showed scant evidence of a positive association between cadmium exposure and BC risk^31^. Compared to no exposure, the summary risk ratio were 1.12 (95% CI 0.80–1.56) and 0.89 (95% CI 0.38–2.14) at 20 µg/day of cadmium intake and 2 µg/g creatinine of cadmium, respectively^31^. Similarly, the SISTER STUDY, including 2587 BC cases with the mean follow-up of 7.4 years, found an HR of 1.1 (95% CI 1.0–1.3) of developing postmenopausal BC when comparing the highest to lowest quintiles of air cadmium levels^32^.

In our previous study based on the same population, we have estimated the risk of BC associated with long-term airborne cadmium exposure, and its effect according to molecular subtype of breast cancer [estrogen receptor negative/positive (ER−/ER+) and progesterone receptor negative/positive (PR−/PR+)]. Our results showed no evidence of an association between airborne exposure to cadmium and risk of overall BC, but suggested an inverse associations for ER− PR− BC^29^. Even if large gaps still remain in the understanding of the potential impact of cadmium on BC development, cadmium may have an effect according to pathological grade, stage or histology subtypes of BC at diagnosis. Since observational studies by BC subtypes are missing, the present study, further explored the association between long-term exposure to cadmium air pollution and risk of BC by stage, grade of differentiation and histological type at diagnosis.

## Results

### Characteristics of the study participants

Differences in cases sociodemographic, reproductive and lifestyle characteristics according to stage are summarized in Table 1. Among the 3924 women who had stage information, the majority were diagnosed at stage I (2370 cases) and at stage II (1216 cases). In contrast, advanced BC was not frequent, only 311 and 27 cases were diagnosed at stages III and IV, respectively. Due to the small number of stage IV BC, stage III and IV were combined in one category (338 cases) to have enough cases for statistical analyses. Women with stage I BC were more likely to be diagnosed older, to be postmenopausal, to be MHT users, to have FHBC, and to have a personal history of benign breast disease. In contrast, women with stages III–IV BC were more likely to be obese (BMI ≥ 30). Also, hormone negative BC (ER− and PR−) were more common among women with advanced stage (III–IV). The distributions of alcohol intake, smoking status, urban/rural status, physical activity, education level, oral contraceptive use, age at menarche, breastfeeding, parity and age at first pregnancy were similar across the three stage groups.

**Table 1:** Demographic and lifestyle characteristics of cases according to the stage of breast cancer at diagnosis in the case–control study nested within the E3N cohort, France, 1990–2008.

Characteristics	Stage I n (%) = 2370 (60.4)	Stage II n (%) = 1216 (31.0)	Stages III–IV n (%) = 338 (8.6)	*P* value
Cumulative airborne cadmium exposure (mg/m^2^), mean ± SD	12.6 ± 58.5	12.9 ± 57.3	14.9 ± 103	0.436
Age at recruitment (years), mean ± SD	49.9 ± 6.1	49.6 ± 6.4	49.5 ± 6.7	0.061
Age at diagnosis (years), mean ± SD	59.6 ± 7.5	59.0 ± 7.8	57.5 ± 8.3	< 0.001
**Alcohol drinking (g/day), n (%)**				
Never	219 (9.2)	102 (8.4)	35 (10.4)	
< 6.7	687 (29.0)	348 (28.6)	88 (26.0)	
≥ 6.7	1,047 (44.2)	522 (42.9)	151 (44.7)	
Missing	417 (17.6)	244 (20.1)	64 (18.9)	0.511
**Body Mass Index (kg/m**^**2**^**), n (%)**				
< 25	2,006 (84.6)	987 (81.2)	271 (80.2)	
25 to < 30	313 (13.2)	185 (15.2)	50 (14.8)	
≥ 30	51 (2.2)	44 (3.6)	17 (5.0)	0.003
**Smoking status, n (%)**				
Never	1270 (53.6)	657 (54.0)	189 (55.9)	
Current	362 (15.3)	187 (15.4)	48 (14.2)	
Former	748 (31.3)	372 (30.6)	101 (29.9)	0.945
**Status of birthplace, n (%)**				
Rural	616 (26.0)	333 (27.4 )	93 (27.5)	
Urban	1,535 (64.8)	766 (63.0)	224 (66.3)	
Missing	219 (9.2)	117 (9.6)	21 (6.2)	0.305
**Physical activity (METs-h/week), n (%)**				
< 25.3	581 (24.5)	303 (24.9)	78 (23.1)	
25.3–37.3	763 (32.2)	357 (29.4)	119 (35.2)	
37.4–56.9	613 (25.9)	332 (27.3)	88 (26.0)	
≥ 57.0	413 (17.4)	224 (18.4)	53 (15.7)	0.444
**Education, n (%)**				
Secondary	292 (12.3)	143 (11.8)	39 (11.5)	
1–2 year university degree	1194 (50.4)	623 (51.2)	176 (52.1)	
≥ 3 year university degree	884 (37.3)	450 (37.0)	123 (36.4)	0.962
**Menopausal status at index date, n (%)**				
Premenopausal	406 (17.1)	254 (20.9)	99 (29.3)	
Postmenopausal	1964 (82.9)	962 (79.1)	239 (70.7)	< 0.001
**Use of oral contraceptives, n (%)**				
No	976 (41.2)	490 (40.3)	135 (39.9)	
Yes	1394 (58.8)	726 (59.7)	203 (60.1)	0.829
**Use of MHT, n (%)**				
No	1924 (81.2)	1003 (82.5)	299 (88.5)	
Yes	446 (18.8)	213 (17.5)	39 (11.5)	0.004
**Parity and age at first pregnancy (AFP), n (%)**				
0	311 (13.1)	168 (13.8)	40 (11.8)	
1–2 & AFP < 30	1190 (50.2)	585 (48.1)	168 (49.7)	
1–2 & AFP ≥ 30	268 (11.3)	143 (11.8)	37 (11.0)	
≥ 3	601 (25.4)	320 (26.3)	93 (27.5)	0.873
**Age at menarche, n (%)**				
< 12	500 (21.1)	275 (22.6)	66 (19.5)	
12–13	1233 (52.0)	637 (52.4)	193 (57.1)	
≥ 14	637 (26.9)	304 (25.0)	79 (23.4)	0.285
**Breastfeeding, n (%)**				
No	1133 (47.8)	566 (46.6)	162 (47.9)	
Yes	1237 (52.2)	650 (53.4)	176 (52.1)	0.760
**Family history of breast cancer, n (%)**				
No	1931 (81.5)	976 (80.3)	293 (86.7)	
Yes	439 (18.5)	240 (19.7)	45 (13.3)	0.026
**History of personal benign breast disease, n (%)**				
No	1643 (69.3)	850 (69.9)	259 (76.6)	
Yes	727 (30.7)	366 (30.1)	79 (23.4)	0.023
**Mammography before inclusion, n (%)**				
No	478 (20.2)	275 (22.6)	103 (30.5)	
Yes	1892 (79.8)	941 (77.4)	235 (69.5)	< 0.001
**ER status, n (%)**				
ER−	345 (14.6)	205 (16.9)	89 (26.3)	
ER+	1590 (67.1)	837 (68.8)	187 (55.3)	
Missing	435 (18.3)	174 (14.3)	62 (18.3)	< 0.001
**PR status, n (%)**				
PR−	647 (27.3)	357 (29.4)	128 (37.9)	
PR+	1225 (51.7)	646 (53.1)	142 (42.0)	
Missing	498 (21.0)	213 (17.5)	68 (20.1)	< 0.001

The analyses were done on the three stages after excluding cases with missing stage information (477 cases).

P values were estimated based on Kruskal–Wallis test for continuous variables and Chi-square test for categorical variables.

*SD* Standard deviation, *MET* Metabolic Equivalent of Task, *MHT* menopausal hormone replacement therapy, Menopausal status at index date: date of diagnosis of the case in the case–control pair, *ER* estrogen receptor, *PR* progesterone receptor.

Analyses comparing the distribution of demographics and risk factors by grade of differentiation of BC at diagnosis showed no substantial differences overall (Supplementary Table S1). Differences across these subtypes were observed for rural/urban status at birth, menopausal status at index date, FHBC, ER and PR status.

Further analyses comparing the distributions of known risk factors for BC according to the histological type are shown in the Supplementary Table S2. Women with invasive tubular carcinoma (ITC) tend to have higher levels of cumulative airborne cadmium exposure as compared to women with other histological types. However, ER and PR + BC were more frequent among women with mixt histology (invasive ductal and lobular carcinoma). Additional analyses comparing the distribution of women’s characteristics with and without BC information (stage, grade of differentiation and histology) are reported in the Supplementary Tables S3, S4, S5, respectively. Compared to the cases with stage information (3924 cases), cases without stage information (477 cases) were less likely to be HRT users, and to have a mammography examination before inclusion. Characteristics were comparable for all other variables (Supplementary Table S3). There was no difference between cases with grade of differentiation information (3433 cases) and cases without grade of differentiation information (968 cases) (Supplementary Table S4). Compared to the cases with histology information (4120 cases), cases without histology information (281 cases) were less likely to be alcohol drinkers, to be physically active, to be oral contraceptive users, and to have a mammography examination before inclusion (Supplementary Table S5).

### Associations between cadmium exposure and BC risk by stage, grade of differentiation, and histological types at diagnosis

Table 2 shows the association between cumulative airborne cadmium exposure index and risk of BC according to the stage in women. Overall, there was no association between cumulative airborne cadmium exposure and BC risk by stage. According to stage, the multivariable adjusted ORs for the fifth versus first quintile were 1.0 (95% CI 0.8–1.3) for stage I BC, 1.1 (95% CI 0.8–1.5) for stage II BC, and 0. 7 (95% CI 0.4–1.2) for stages III–IV BC (Table 2). There was no association between cadmium and risk of stage of BC among both premenopausal and postmenopausal women (Supplementary Table S6).

**Table 2:** Odds ratio and 95% confidence intervals (OR, 95% CI) for the association of quintiles of the cumulative airborne cadmium exposure with risk of breast cancer according to breast cancer stage in the case–control study nested within the E3N cohort, France, 1990–2008.

Cumulative airborne cadmium exposure (mg/m^2^)	n cases/controls	OR (95% CI)^a^	*P* trend	*P* likelihood	*P* heterogeneity
**Stage I**					
≤ 0.072	454/481	Ref			
> 0.072–0.767	491/464	1.1 (0.9–1.4)			
> 0.767–2.822	491/462	1.2 (0.9–1.4)			
> 2.822–11.07	470/492	1.1 (0.9–1.3)			
> 11.07	464/471	1.0 (0.8–1.2)	0.882	0.502	
**Stage II**					
≤ 0.072	257/242	Ref			
> 0.072–0.767	217/265	0.8 (0.6–1.1)			
> 0.767–2.822	253/242	1.1 (0.8–1.4)			
> 2.822–11.07	236/237	0.9 (0.7–1.3)			
> 11.07	253/230	1.1 (0.8–1.5)	0.296	0.208	
**Stages III–IV**					
≤ 0.072	78/63	Ref			
> 0.072–0.767	69/70	0.8 (0.5–1.4)			
> 0.767–2.822	66/85	0.6 (0.4–1.1)			
> 2.822–11.07	63/55	0.8 (0.4–1.4)			
> 11.07	62/64	0.7 (0.4–1.3)	0.253	0.470	0.455

^a^Multivariable models were adjusted for physical activity, tobacco smoking status, alcohol intake, level of education, body mass index, age at menarche, age at first full-term pregnancy, parity, breastfeeding, oral contraceptive use, menopausal hormone replacement therapy use, status of birthplace, previous family history of breast cancer and personal history of benign breast disease.

P likelihood: P-values from likelihood ratio test comparing the statistically significance of the global effect of the quintiles.

P heterogeneity: comparing heterogeneity of associations across breast cancer stage at diagnosis.

The association between cumulative airborne cadmium exposure index and risk of BC by grade of differentiation is shown in Table 3. Overall, no associations were observed between levels of cumulative airborne cadmium exposure index and risk of BC by grade of differentiation at diagnosis. The ORs comparing the fifth quintile to the reference category (first) = 1.2 (95% CI 0.7–1.9) for grade 1 BC, 1.1 (95% CI 0.8–1.5) for grade 2 BC and 0.9 (95% CI 0.7–1.2) for grade 3 BC. There was no heterogeneity of the results by grade of differentiation.

**Table 3:** Odds ratio and 95% confidence intervals (OR, 95% CI) for the association of quintiles of the cumulative airborne cadmium exposure with breast cancer risk by grade of differentiation in the case–control study nested within the E3N cohort, France, 1990–2008.

Cumulative airborne cadmium exposure (mg/m^2^)	n cases/controls	OR (95% CI)^a^	*P* trend	*P* likelihood	*P* heterogeneity
**Grade 1**					
≤ 0.072	101/104	Ref			
> 0.072–0.767	116/114	1.2 (0.8–1.8)			
> 0.767–2.822	116/108	1.2 (0.8–1.8)			
> 2.822–11.07	107/122	1.0 (0.6–1.5)			
> 11.07	108/100	1.2 (0.7–1.9)	0.861	0.760	
**Grade 2**					
≤ 0.072	262/264	Ref			
> 0.072–0.767	251/251	1.0 (0.8–1.3)			
> 0.767–2.822	248/261	1.0 (0.8–1.3)			
> 2.822–11.07	237/236	1.0 (0.8–1.4)			
> 11.07	265/251	1.1 (0.8–1.5)	0.516	0.972	
**Grade 3**					
≤ 0.072	321/323	Ref			
> 0.072–0.767	318/335	1.0 (0.8–1.2)			
> 0.767–2.822	345/306	1.1 (0.9–1.4)			
> 2.822–11.07	335/332	1.0 (0.8–1.2)			
> 11.07	302/325	0.9 (0.7–1.2)	0.481	0.632	0.934

^a^Multivariable models were adjusted for physical activity, tobacco smoking status, alcohol intake, level of education, body mass index, age at menarche, age at first full-term pregnancy, parity, breastfeeding, oral contraceptive use, menopausal hormone replacement therapy use, status of birthplace, previous family history of breast cancer and personal history of benign breast disease.

P likelihood: P-values from likelihood ratio test comparing the statistically significance of the global effect of the quintiles.

P heterogeneity: comparing heterogeneity of associations across breast cancer grade at diagnosis.

With respect to the histological types, we found a suggestive evidence that the association between cadmium and BC risk varied by histology (Table 4). Cadmium was associated with higher risk of ITC with the adjusted OR for the fifth versus first quintile being 3.4 (95% CI 1.1–10.7). No relationships were found for invasive ductal carcinoma (IDC), invasive lobular carcinoma (ILC) or mixt BC. Results of the additional cubic splines modelling using four knots with the minimum value as the reference category, suggested a dose–response relation between cumulative airborne BaP exposure and ITC risk (Supplementary Fig. S1). Cubic splines dose–response assessments were also reported for all BC subtypes (histology: IDC, ILC or mixt BC, stage: I to III–IV, and grade: 1 to 3) in the Supplementary Figs. S2–S9, respectively. However, there was no relationship between these subtypes and cadmium exposure. In sensitivity analyses, the multivariable risk estimates did not substantially change after further adjustment for mammographic examination before inclusion (Supplementary Table S7). Similarly, using the mean annual cadmium exposure instead of cumulative airborne cadmium exposure showed similar findings (Supplementary Table S8). Further complete-case analysis (exclusion of cases with missing values), yielded ORs comparable to the main analyses using imputation.

**Table 4:** Odds ratio and 95% confidence intervals (OR, 95% CI) for the association of quintiles of the cumulative airborne cadmium exposure with risk of breast cancer according to the histological type in the case–control study nested within the E3N cohort, France, 1990–2008.

Cumulative airborne cadmium exposure (mg/m^2^)	n cases/controls	OR (95% CI)^a^	*P* trend	*P* likelihood	*P* heterogeneity
**Invasive ductal carcinoma**					
≤ 0.072	580/608	Ref			
> 0.072–0.767	608/595	1.1 (0.9–1.3)			
> 0.767–2.822	614/586	1.1 (0.9–1.3)			
> 2.822–11.07	572/573	1.1 (0.9–1.3)			
> 11.07	566/578	1.0 (0.8–1.2)	0.929	0.695	
**Invasive lobular carcinoma**					
≤ 0.072	145/131	Ref			
> 0.072–0.767	110/140	0.8 (0.6–1.2)			
> 0.767–2.822	136/130	1.0 (0.7–1.4)			
> 2.822–11.07	150/142	1.0 (0.7–1.5)			
> 11.07	135/133	1.0 (0.6–1.4)	0.790	0.706	
**Invasive tubular carcinoma**					
≤ 0.072	18/28	Ref			
> 0.072–0.767	25/28	1.71 (0.6–4.7)			
> 0.767–2.822	32/29	1.53 (0.6–4.0)			
> 2.822–11.07	26/21	2.55 (0.8–8.3)			
> 11.07	31/26	3.4 (1.1–10.7)	0.034	0.257	
**Ductal-lobular BC**					
≤ 0.072	25/21	Ref			
> 0.072–0.767	21/15	1.3 (0.4–4.4)			
> 0.767–2.822	19/22	0.8 (0.2–2.6)			
> 2.822–11.07	18/24	0.5 (0.2–1.7)			
> 11.07	26/27	1.0 (0.3–3.6)	0.436	0.648	0.347

^a^Multivariable models were adjusted for physical activity, tobacco smoking status, alcohol intake, level of education, body mass index, age at menarche, age at first full-term pregnancy, parity, breastfeeding, oral contraceptive use, menopausal hormone replacement therapy use, status of birthplace, previous family history of breast cancer and personal history of benign breast disease.

P likelihood: P-values from likelihood ratio test comparing the statistically significance of the global effect of the quintiles.

P heterogeneity: comparing heterogeneity of associations across histological type of breast cancer.

## Discussion

To the best of our knowledge, this is the first epidemiological study exploring the association between airborne cadmium exposure and risk of BC by stage, grade of differentiation, and by histological type. The results of this nested case–control study do not support the hypothesis that cumulative airborne exposure to cadmium increases the risk of advanced stage BC at diagnosis. Further analyses by grade of differentiation of BC at diagnosis also showed no evidence of an association between airborne cadmium and risk of BC by grade. In contrast, we found an increased risk of ITC BC, suggesting that the association of cadmium air pollution with BC may differ by histological types. These women also had significantly higher levels of cadmium as compared to women with other histological subtypes of BC.

Few epidemiological studies have reported an increased risk of overall BC associated with higher cadmium levels in urine^33,34^ or dietary cadmium^35,36^. A 2016 random effect meta-analysis reported that higher level of urinary cadmium was associated with a higher risk of BC, pooled OR of the highest versus lowest quantile was 2.24 (95% CI 1.49–3.35)^37^. A more recent meta-analysis did not support an association between cadmium and overall BC, although they suggested marginal positive relation between dietary cadmium intake and BC^31^. Only one study has assessed the association between cadmium and risk of BC by histological subtype, and found an increased risk of ductal BC with an adjusted OR of 1.1 (95% CI 0.89–1.58) in the intermediate and 1.53 (95% CI 1.15–2.04) in the highest category of urinary cadmium as compared to the lowest tertile. However this study did not investigate tubular BC^38^.

The results of the present study add to the current evidence that risk may vary across histological BC types, and suggest a role of cadmium in the etiology of ITC. However, although the cubic splines modelling suggested a potential dose response relation between cadmium exposure and ITC, our results should be interpreted with caution, due to the small numbers of ITC and the possibility of false positive findings as a result of multiple testing.

Conversely, there is an increasing body of laboratory evidence supporting that cadmium promotes BC cell growth, particularly metastasis^15,39^. In our study, although the cumulative airborne cadmium exposure index was higher in advanced stages of BC, the multivariable analyses showed no statistically significant association. Overall, our results were not consistent with the findings from several experimental studies reporting that BC risk associated with cadmium may vary according to the stage^22,40^. Likewise, Peng et al. reported that high cadmium exposure was observed in advanced stages of BC, raising the question of a possible role in BC development and progression^41^^.^ Also, the lack of association between airborne cadmium and grade of differentiation of BC observed in this study is not in line with mechanistic evidence suggesting that cadmium may play a role in the differentiation of BC cells and tissues^27^. The inconsistency observed between our results and experimental findings is likely to be explained by the size of some of the subgroups that was too small to provide an accurate estimate of the effect, particularly for the analyses by stage. Further epidemiological studies with more cases by stage, and grade of BC are warranted to evaluate whether cadmium may be associated to specific clinico-pathological subtypes of BC. Also, in our population, even if women were chronically exposed for long periods, they were generally exposed to low doses that may not reflect the short-term high cadmium doses in experimental studies shown to induce aggressiveness and transformation of non-cancerous human mammary epithelial cells^23,42,43^.

The exact mechanisms linking cadmium and BC development are still unclear. Several potential mechanisms have been proposed, involving both estrogen mediated pathways and ER independent mechanisms. Cadmium has been shown to interfere with a number of normal estrogen-sensitive pathways. In particular, cadmium can interact with the hormone-binding domain of ER^44^ to regulate several genes and transcription factors involved in BC cell growth and proliferation^45^. Recently, Bloomfield demonstrated that chronic cadmium exposure, even at low levels, can increase the malignancy of BC cells by decreasing their dependency on ERα and increasing the adaptability of the cancer cells^46^. Additionally, cadmium can promote the development of cancer through several ER independent mechanisms. Cadmium has been shown to activate the production of reactive oxygen species (ROS) and reduce the anti-oxidative defenses in breast tumor cells, one of the major mechanisms of breast carcinogenesis^47^. Furthermore, cadmium induces genotoxic effects, including DNA modifications due to cadmium-induced oxidative damage^48^. Also, cadmium has been found to alter DNA repair and cause genomic instability^48^.

Strengths of our study include the prospective design of the E3N cohort study and the high quality information collected. One of the most important strength of our study is the accurate geocoding of the residential history to reconstruct exposure variation over time, resulting from changes in source emissions over the study period as well as from the subjects’ residential moves. Cadmium sources emission estimates were compared to direct stack measurements over time, and showed good overall performance^49^. Unlike our study, the majority of epidemiological studies considered exposure at a single point in time under the assumption that a single measure represents a proxy for that exposure over a longer time. Another essential strength of our study is the availability of detailed information on individual reproductive and lifestyle factors allowing extensive consideration of potential confounding, particularly education level, tobacco smoking, BMI, and urban rural status factors. As discussed in our previous study on airborne and BC risk^29^, a further important strength is the reliability of our GIS-based metric. Furthermore, the assessment of the performance of the GIS-based metric for cadmium exposure estimates demonstrated a strong concordance with the dispersion model exposure estimate (SIRANE) at different locations and periods of time^50^. The GIS-based metric demonstrated its ability to reliably characterize long-term environmental cadmium exposures of study subjects^51^, and has been applied in two previous studies^29,52^. Limitations of our study comprise the lack of earlier residential history (before 1990), historical airborne cadmium exposure estimates before inclusion into the cohort in 1990^53^, as well as direct comparison between the GIS-based metric and biological measurements. This left truncation of exposure data has been reported to lead to a loss of accuracy of exposure estimates and may be associated with potential overestimation or underestimation of the relation between exposure and the risk of disease^54,55^. Also, a larger proportion of inhaled cadmium is retained by the body as compared to ingested cadmium (30–60% versus 2–6%)^21^. The missing information on stage (10.8% of women), grade of differentiation (22.0%) and histology (6.4%) may be a limitation in the present study. However, further analyses comparing the characteristics distribution of women with and without BC information (stage, grade of differentiation and histology) showed no important differences. Lastly, the lack of full addresses for the 3rd (Q3) and 4th (Q4) questionnaires (only postal codes available) might be a limitation in the reliability of exposure assessment, nevertheless we doubt that this could affect the associations between cadmium exposure and BC subtype risk, since we used an explicit imputation to recover the complete addresses.

Our study does not support the hypothesis that cadmium air pollution exposure may have a role in the risk of advanced stage BC and in the differentiation of breast tumor cells, suggested in experimental studies. The observed positive association between cadmium and ITC BC should be interpreted with caution.

## Methods

### Study design and participants

E3N (Etude Epidémiologique auprès de femmes de la Mutuelle Générale de l'Education Nationale) is an ongoing prospective cohort study involving 98,995 French women, established in 1990 to investigate risk factors for cancer and severe chronic conditions in women^56^. Participants were recruited between June 1990 and November 1991 among women aged 40–65 years, living in France and insured with the MGEN, a national health insurance plan covering people working within the French education system and their families, and have been biennially followed-up with self-administered mailed questionnaires. At recruitment, participants filled in a self-administered questionnaire, which included items relative to lifestyle and reproductive factors, anthropometry, past medical history, and family history of cancer. To date, twelve questionnaires have been sent to the participants (participation rate at each questionnaire ~ 80.0%). Between 1994 and 1998, participants were invited to give a blood specimen, resulting in the collection from 25,000 women, and saliva samples were later collected from an additional 47,000 women. Occurrence of cancer was self-reported in each questionnaire, and a small number of cancers were further identified from the insurance files or information on causes of death obtained from the National Service on Causes of Deaths (CépiDC-Inserm). The pathology report for confirming diagnosis of the primary outcome (invasive BC) was obtained for 93.0% of declared cases. The addresses of the subjects selected for the study have been reported in the baseline questionnaire (1990) and in the 5th to 9th follow-up questionnaires (years 1997, 2000, 2002, 2005, and 2008). In the 3rd and 4th follow-up questionnaires (years 1993 and 362 only postal codes of participants were reported. For missing addresses during follow-up (until index date), we used decision rules to assign addresses. For the 3rd (Q3) and 4th (Q4) questionnaires, for which only postal codes were recorded, we recovered the complete addresses (number, street, postal code and town) from questionnaires Q1 and Q5. If the complete address of Q1 and Q5 were identical and the postal codes of Q3 and Q4 corresponded to the postal code of Q1 and Q5, we assumed that the woman had not moved between Q1 and Q5 and we assigned to Q3 and Q4 the address of Q1. When the addresses of Q1 and Q5 were different, the postal codes of Q3 and Q4 were compared to the postal codes of Q1 and Q5 ones. If there were equal to Q1, we assigned the address of Q1. If there were equal to Q5, we assigned the address of Q5. In addition, participants’ place of birth (postal code and municipality) was obtained from the first questionnaire and assigned an urban/rural status based on data from the closest national census^57^. Informed consent was obtained from all participants and the study was approved by the French National Commission for Data Protection and Privacy (CNIL) Ethics committee. The E3N cohort is registered in the French epidemiology database “Portail Epidémiologie France Health databases” [CNIL n°327346V 13, CPP (03/12/2008)].

### The nested case control study design

A nested case–control study was designed among women of the E3N cohort who had completed their home address at baseline, lived in the metropolitan French territory during the follow-up time, and had never been diagnosed with any cancer at baseline. Details of this study have been described elsewhere^29^. After excluding women with phyllodes tumors, a total of 5382 incident cases of primary invasive BC were identified during the follow-up 1990 to 2008. As the proportion of false-positive self-reports was low (< 5%), we also included participants who reported a BC diagnosis for whom pathology reports had not been obtained.

From these, we excluded women with missing addresses (including women with more than one missing address and those for whom it was impossible to retrieve addresses, N = 981 cases). For each BC case, one control was randomly selected using incidence density sampling, i.e. among cohort participants at risk of BC at the time of case diagnosis, using the follow-up time since inclusion into the cohort as time axis. In order to best select appropriate controls according to the planned studies, two complementary groups of cases were set, according to availability of a biological sample (blood or saliva), for the first group of cases (with a blood sample), controls were matched to cases on the department of residence, age (± 1 year), date (± 3 months) and menopausal status at blood collection. Controls for the second group (without blood sample) were matched on the same criteria but collected at baseline, and additionally matched on the existence or not of a saliva sample. To be noted, false positives BC cases were not excluded in the control group, as they are not BC. Finally, the nested case–control study involved 4401 women diagnosed with a primary invasive BC and 4401 matched controls with complete information on home address at baseline^29^.

### Assessment of staging, grading, histology and other covariates

Information on tumor-node-metastasis (TNM) stage was extracted from pathological reports or any other medical document (such as bone-scan, magnetic resonance (MRI) or X-ray radiography reports). Of the 4401 BC cases, a total of 3924 (89.2%) cases had stage information. Information on the grade of differentiation and histological subtype at diagnosis was collected based on pathological reports and available for 3433 (78.0%) and 4120 (93.6%) BC cases, respectively. Data on established BC risk factors and other potential confounding factors were obtained from the E3N self-administered questionnaires at baseline. Information collected at baseline on smoking, anthropometry (height, weight), physical activity, diabetes, hypertension, benign breast disease, gynecological follow-up, family history of BC (FHBC), education level, age at menarche and at menopause, use of exogenous hormones, number of children, age at first full-term pregnancy (AFP), and breastfeeding^56^. Follow-up questionnaires were sent every 2–3 years thereafter. Daily alcohol intake (g/day) was estimated from the validated E3N self-administered diet history questionnaire (DHQ) in 1993. Physical activity was converted into metabolic equivalent task-hour per week (MET-h/w).

### Assessment of long-term exposure to airborne cadmium

The method employed to estimate airborne cadmium exposure at the individual residential address level have has been previously described in detail^51,53,58^ and applied in two previous studies^29,59^. Briefly, the residential history of the women, from their enrolment in the E3N cohort until the index date (BC diagnosis for cases, date of diagnosis of the case in the case–control pair for controls) was used to estimate atmospheric exposure to cadmium, within a Geographic Information System (GIS). A GIS-based metric was developed and calibrated using a set of parameters (local meteorological data, characteristics of industrial sources, e.g. emission intensity and stack height)^51^. Cadmium concentrations estimated by the SIRANE Gaussian dispersion model^60^ was used as a reference for the development of the present GIS-based metric. SIRANE is an urban dispersion model that integrates a specific module to simulate pollutant dispersion within a built environment, considering local meteorological conditions and geometry of the streets^50,61^.

Firstly, a detailed retrospective inventory of industrial cadmium emitting sources over the entire metropolitan France between 1990 and 2008 was performed^53^. Sources of emissions were assessed using emission factors from the OMINEA (Organization and Methods of the National Inventories of the Atmospheric Releases in France)^62^ and the EMEP (European Monitoring and Evaluation Program)^63^ databases. Overall, 2700 cadmium sources were inventoried over the French national territory from 1990 to 2008^53^.

The participants residential history from 1990 to 2008 and inventoried cadmium emitting sources were geocoded (X and Y coordinates, addresses) using the ArcGIS Software (ArcGIS Locator version 10.0, Environmental System Research Institute—ESRI, Redlands, CA, USA) and its reference street network database, BD Adresse^®^, from the National Geographic Institute (IGN)^58^.

To classify the study subjects according to their airborne cadmium exposure, the annual airborne cadmium exposure index (AACEI) was estimated using the following GIS-based metric:

$$\mathrm{AACEI }\left({\mathrm{mg}/\mathrm{m}}^{2}\right)=\sum_{\mathrm{j}}^{\mathrm{J}}\sum_{\mathrm{i}}^{\mathrm{I}}\mathrm{tj}\times \frac{1}{{{\mathrm{d}}^{2}}_{\mathrm{ij}}}\times {\mathrm{EI}}_{\mathrm{i}}\times {\mathrm{F}}_{\mathrm{i}}\times {\left(\frac{{\mathrm{h}}_{\mathrm{median}}}{{\mathrm{h}}_{\mathrm{i}}}\right)}^{a},$$

where j was the place of residence (j = 1,…,J); i was the industrial source (i = 1,…,I), EI was the source annual cadmium emission intensity (in kg/year); t was the emission period duration (in year); d was the residence-to-source distance (in m); Fi was the factor accounting for the weighted contribution of wind direction from the industrial source *i* to the participant’s residence *j*; h_i_ was the stack height (in m); h_median_ was the median value of the other sources’ stack height (in m) in a 10 km buffer, and was taken into account only when h_i_ was greater than 90 m.

The exposure to cadmium was computed for each individual and for each calendar year. For each individual, their cumulative airborne cadmium exposure index was calculated by cumulating their AACEI from their entry into the cohort to their index date. The cumulative airborne cadmium exposure index was then expressed from kg/m^2^ to mg/m^2^^29^.

### Statistical analyses

Kruskal–Wallis and Chi-square tests were used to assess BC cases characteristics differences according to stage, grade of differentiation, histological type with regard to continuous and categorical variables, respectively.

Conditional logistic regression models were used to estimate odds ratios (OR) and their 95% confidence intervals (95% CI) for risk of BC associated with cadmium exposure. Models were conditioned on the matching factors including date of blood collection or of the return of the first questionnaire, age, department of residence, menopausal status at blood collection or at baseline, and existence of a biological sample (blood, saliva, none). Two adjusted models were considered to account for predefined variables recognized as confounding and risk factors for BC. Using a directed acyclic graph to identify the confounding variables, the first model was adjusted for physical activity (< 25.3, 25.3–37.3, 37.4–56.9, and ≥ 57.0 METs-h/week), alcohol intake (never, < 6.7, ≥ 6.7 g/day), level of education (secondary, 1 to 2-year university degree, ≥ 3 year-university degree), BMI (< 25, 25 to  < 30, and ≥ 30 kg/m^2^), age at menarche (< 12, 12–13, and ≥ 14 years), parity and AFP (0, 1–2 children & AFP < 30 years, 1–2 children & AFP ≥ 30 years, and ≥ 3 children), breastfeeding (ever, never), oral contraceptive use (ever, never), MHT (ever, never), status of birthplace (rural, urban)^9,57,59^ and smoking status (never, current, and former). In the second multivariable model, we further adjusted for previous FHBC (yes, no) and history of personal benign breast disease (yes, no). Since there was no difference in the OR estimates between the two models, we only reported the results of the fully adjusted models in the main manuscript. For contraceptive and MHT variables, we considered the values collected in the last questionnaire before the date of diagnosis in cases, whereas all other adjustment variables were taken at E3N baseline questionnaire.

For covariates with less than 5.0% missing data, the latter were imputed by their modal or median value of the control population; and for variables with more than 5.0% of missing data (only alcohol intake and rural urban status at birth), a category of missing data was created.

Statistical analyses for quintiles of the cumulative airborne cadmium exposure index were performed by stage, grade of differentiation, and histological subtype of BC at diagnosis using the first quintile as the reference value. Quintile cut-points of the cumulative levels were based on the distribution in control subjects. For each variable, the *P* for linear trend was the P-value associated with the regression coefficient of the categorical variable used as continuous. The statistically significance of the global effect of the quintiles of the cumulative airborne cadmium was derived from the likelihood ratio test comparing the models including and excluding terms for quintiles. Heterogeneity of associations across BC subtypes was assessed using polytomous logistic regression and *P* values for heterogeneity were derived from Wald tests^64^.

For sensitivity analyses, we repeated our analyses using the mean of the annual airborne cadmium exposure index (from entry into the cohort to the index date) of the cadmium exposure instead of the cumulative exposure. Additional adjustments for the mammographic examination before inclusion (yes, no) was also done. To assess whether the missing information on BC (stage, grade of differentiation and histology) may affect associations, we further performed supplementary analyses comparing the characteristics distribution of women with and without BC information (stage, grade and histology). Also, to assess whether imputation of missing values affected observed associations, we repeated the analyses after excluding participants with missing values in any covariate (complete-case analysis).

Cubic splines^65^, with four knots placed at 5th, 35th, 65th, and 95th percentiles of the cumulative exposure to airborne cadmium distribution^66^ were performed to explore the non-linearity of the relationship between cadmium and each BC subtype risk.

All statistical tests were two-sided and a threshold of *P* values < 0.05 were considered statistically significant. All analyses were performed using STATA version 14 (College Station, Texas, USA).

### Ethics approval and consent to participate

We confirm that all methods were carried out in accordance with relevant guidelines and regulations. The study was approved by the Ethics committee “the French National Commission for Data Protection and Privacy (CNIL)”, and informed consent was obtained from all individual participants included in the study.

### Consent for publication

All of the authors have read and approved the article.

## Supplementary Information

Supplementary Information.

## Abbreviations

AACEI: Annual airborne cadmium exposure index
AFP: Age at first full-term pregnancy
BMI: Body mass index
BC: Breast cancer
CIs: Confidence intervals
CNIL: Commission for data protection and privacy
DHQS: Diet history questionnaires
E3N: Etude Epidémiologique auprès de femmes de la Mutuelle Générale de l'Education Nationale
ER: Estrogen receptor
EPIC: European Prospective Investigation into Cancer and Nutrition
EMEP: European Monitoring and Evaluation Program
EDC: Endocrine-disrupting-chemicals
FHBC: Family history of breast cancer
GIS: Geographic information system
IDC: Invasive ductal carcinoma
ILC: Invasive lobular carcinoma
ITC: Invasive tubular carcinoma
IARC: International Agency for Research on Cancer
MHT: Menopausal hormone therapy
MET: Metabolic equivalent task
IGN: National Geographic Institute
ORs: Odds ratios
PR: Progesterone receptor
OMINEA: Organization and Methods of the National Inventories of the Atmospheric Releases
SD: Standard deviation
TNM: Tumor-node-metastasis
TCDD: Tetrachlorodibenzo-p-dioxin
wκ: Weighted kappa
WHO: World Health Organization
