# Supplementary material for: Exposure to airborne cadmium and breast cancer stage, grade and histology at diagnosis: findings from the E3N cohort study
Source: Sci Rep. 2021 Nov 29;11:23088. doi: 10.1038/s41598-021-01243-0 (PMC8630221; doi:10.1038/s41598-021-01243-0)
Supplement: Supplementary file 1 — Supplementary Information. [file 41598_2021_1243_MOESM1_ESM.docx]

**Supplementary Tables and Figures**

**Exposure to airborne cadmium and breast cancer stage, grade and histology at diagnosis: Findings from the E3N cohort study**

Amina Amadou ^1 2^, Delphine Praud ^1 2^, Thomas Coudon ^1 2 3^, Aurélie M N Danjou ^1^, Elodie Faure ^5^, Floriane Deygas ^1 2^, Lény Grassot ^1 2^, Karen Leffondré ^4^, Gianluca Severi ^5 6^, Pietro Salizzoni ^3^, Francesca Romana Mancini ^5*^, and Béatrice Fervers ^1 2 *^

^1^ Département Prévention Cancer Environnement, Centre Léon Bérard, Lyon, France

^2^ Inserm UA 08 Radiations : Défense, Santé, Environnement, Lyon, France

^3^ Ecole Centrale de Lyon, INSA Lyon, Université Claude Bernard Lyon 1, Ecully, France

^4^ Université de Bordeaux, ISPED, Inserm U1219, Bordeaux Population Health Center, Bordeaux, France

^5^ Paris-Saclay University, UVSQ, Univ. Paris-Sud, Inserm, Gustave Roussy, Team "Exposome and Heredity", CESP, 94805, Villejuif, France

^6^ Departement of Statistics, Computer Science and Applications (DISIA), University of Florence, Italy

**Supplementary Tables and Figures**

Supplementary Table S1 online: Demographic and lifestyle characteristics of cases according to grade of differentiation of breast cancer at diagnosis in the case-control study nested within the E3N cohort, France, 1990-2008.

Supplementary Table S2 online**:** Demographic and lifestyle characteristics of cases according to histological type of breast cancer at diagnosis in the case-control study nested within the E3N cohort, France, 1990-2008.

Supplementary Table S3 online: Distribution of baseline demographic and risk factors of breast cancer between women without and with stage information in the case-control study nested within E3N cohort, France, 1990-2008.

Supplementary Table S4 online: Distribution of baseline demographic and risk factors of breast cancer between women without and with grade information in the case-control study nested within E3N cohort, France, 1990-2008.

Supplementary Table S5 online: Distribution of baseline demographic and risk factors of breast cancer between women without and with histology information in the case-control study nested within E3N cohort, France, 1990-2008.

Supplementary Table S6: Odds ratio and 95% confidence intervals (OR, 95% CI) for the association between mean airborne cadmium exposure and risk of breast cancer according to stage in pre- and post-menopausal women at index date: The case-control study nested within the E3N cohort, France, 1990-2008.

Supplementary Table S7: Sensitivity analyses of the multivariable model additionally adjusted for mammographic examination before inclusion: Odds ratio and 95% confidence intervals (OR, 95% CI) for the association of quintiles of the cumulative airborne cadmium exposure with risk of breast cancer according to breast cancer stage in the case-control study nested within the E3N cohort, France, 1990-2008.

Supplementary Table S8 online: Odds ratio and 95% confidence intervals (OR, 95% CI) for the association of quintiles of the mean airborne cadmium exposure with risk of breast cancer according to the stage in the case-control study nested within the E3N cohort, France, 1990-2008.

Supplementary Fig.S1 online: Cubic spline modelling of the relationship between cumulative airborne cadmium exposure and risk of invasive tubular breast cancer in the case-control study nested within the E3N cohort, France, 1990-2008.

Supplementary Fig.S2: Cubic spline modelling of the relationship between cumulative airborne cadmium exposure and risk of invasive ductal breast cancer in the case-control study nested within the E3N cohort, France, 1990-2008.

Supplementary Fig.S3: Cubic spline modelling of the relationship between cumulative airborne cadmium exposure and risk of invasive lobular breast cancer in the case-control study nested within the E3N cohort, France, 1990-2008.

Supplementary Fig.S4: Cubic spline modelling of the relationship between cumulative airborne cadmium exposure and risk of invasive mixt (ductal lobular) breast cancer in the case-control study nested within the E3N cohort, France, 1990-2008.

Supplementary Fig.S5: Cubic spline modelling of the relationship between cumulative airborne cadmium exposure and risk of stage I breast cancer in the case-control study nested within the E3N cohort, France, 1990-2008.

Supplementary Fig.S6: Cubic spline modelling of the relationship between cumulative airborne cadmium exposure and risk of stage II breast cancer in the case-control study nested within the E3N cohort, France, 1990-2008.

Supplementary Fig.S7: Cubic spline modelling of the relationship between cumulative airborne cadmium exposure and risk of stage III-IV breast cancer in the case-control study nested within the E3N cohort, France, 1990-2008.

Supplementary Fig.S8: Cubic spline modelling of the relationship between cumulative airborne cadmium exposure and risk of grade 1 breast cancer in the case-control study nested within the E3N cohort, France, 1990-2008.

Supplementary Fig.S9: Cubic spline modelling of the relationship between cumulative airborne cadmium exposure and risk of grade 2 breast cancer in the case-control study nested within the E3N cohort, France, 1990-2008.

Supplementary Fig.S9: Cubic spline modelling of the relationship between cumulative airborne cadmium exposure and risk of grade 3 breast cancer in the case-control study nested within the E3N cohort, France, 1990-2008.

**Supplementary** **Table S1**: Demographic and lifestyle characteristics of cases according to grade of differentiation of breast cancer at diagnosis in the case-control study nested within the E3N cohort, France, 1990-2008

| Characteristics | Grade 1  n (%)=548 (16.0%) | Grade 2  n (%)=1,263 (36.8%) | Grade 3  n (%)=1,621 (47.2%) | *P* value |
| --- | --- | --- | --- | --- |
| Cumulative airborne cadmium exposure (mg/m^2^), mean ± SD | 19.0 ± 120.8 | 13.0 ± 61.6 | 10.4 ± 31.9 | 0.885 |
| Age at recruitment (years), mean ± SD | 49.5 ± 5.9 | 49.8 ± 6.3 | 49.8 ± 6.4 | 0.770 |
| Age at diagnosis (years), mean ± SD | 59.1 ± 7.3 | 59.0 ± 7.6 | 60.0 ± 7.7 | <0.001 |
| Alcohol drinking (g/day), n (%) |  |  |  |  |
| Never | 57 (10.4) | 110 (8.7) | 144 (8.9) |  |
| < 6.7 | 150 (27.4) | 364 (28.8) | 468 (28.9) |  |
| ≥ 6.7 | 254 (46.3) | 529 (41.9) | 731 (45.1) |  |
| Missing | 87 (15.9) | 260 (20.6) | 278 (17.1) | 0.101 |
| Body Mass Index (kg/m²), n (%) |  |  |  |  |
| < 25 | 475 (86.7) | 1,046 (82.8) | 1,323 (81.6) |  |
| 25 - <30 | 63 (11.5) | 177 (14.0) | 252 (15.6) |  |
| ≥ 30 | 10 (1.8) | 40 (3.2) | 46 (2.8) | 0.073 |
| Smoking status, n (%) |  |  |  |  |
| Never | 298 (54.4) | 697 (55.2) | 871 (53.7) |  |
| Current | 79 (14.4) | 180 (14.2) | 247 (15.2) |  |
| Former | 171 (31.2) | 386 (30.6) | 503 (31.0) | 0.930 |
| Status of birthplace, n (%) |  |  |  |  |
| Rural | 128 (23.4) | 359 (28.4) | 438 (27.0) |  |
| Urban | 382 (69.7) | 804 (63.5) | 1,023 (63.1) |  |
| Missing | 38 (6.9) | 102 (8.1) | 160 (9.9) | 0.020 |
| Physical activity (METs-h/week), n (%) | |  |  |  |
| < 25.3 | 128 (23.4) | 338 (26.8) | 379 (23.4) |  |
| 25.3 - 37.3 | 182 (33.2) | 372 (29.4) | 514 (31.7) |  |
| 37.4 - 56.9 | 140 (25.5) | 340 (26.9) | 423 (26.1) |  |
| ≥ 57.0 | 98 (17.9) | 213 (16.9) | 305 (18.8) | 0.266 |
| Education, n (%) |  |  |  |  |
| Secondary | 73 (13.3) | 155 (12.3) | 194 (12.0) |  |
| 1- to 2-year university degree | 260 (47.5) | 646 (51.1) | 834 (51.4) |  |
| ≥ 3 year university degree | 215 (39.2) | 462 (36.6) | 593 (36.6) | 0.581 |
| Menopausal status, n (%) |  |  |  |  |
| Premenopausal | 98 (17.9) | 272 (21.5) | 254 (15.7) |  |
| Postmenopausal | 450 (82.1) | 991 (78.5) | 1,367 (84.3) | <0.001 |
| Use of oral contraceptives, n (%) | |  |  |  |
| No | 220 (40.1) | 533 (42.2) | 645 (39.8) |  |
| Yes | 328 (59.9) | 730 (57.8) | 976 (60.2) | 0.406 |
| Use of MHT, n (%) |  |  |  |  |
| No | 446 (81.4) | 1,056 (83.6) | 1,318 (81.3) |  |
| Yes | 102 (18.6) | 207 (16.4) | 303 (18.7) | 0.242 |
| Parity & Age at First Pregnancy (AFP), n (%) | |  |  |  |
| 0 | 74 (13.5) | 171 (13.5) | 202 (12.5) |  |
| 1-2 & AFP < 30 | 282 (51.5) | 611 (48.4) | 822 (50.7) |  |
| 1-2 & AFP ≥ 30 | 53 (9.7) | 147 (11.6) | 182 (11.2) |  |
| ≥ 3 | 139 (25.3) | 334 (26.4) | 415 (25.6) | 0.754 |
| Age at menarche, n (%) |  |  |  |  |
| < 12 | 118 (21.5) | 265 (21.0) | 354 (21.8) |  |
| 12 - 13 | 279 (50.9) | 675 (53.4) | 850 (52.4) |  |
| ≥ 14 | 151 (27.6) | 323 (25.6) | 417 (25.7) | 0.851 |
| Breastfeeding, n (%) |  |  |  |  |
| No | 261 (47.6) | 605 (47.9) | 760 (46.9) |  |
| Yes | 287 (52.4) | 658 (52.1) | 861 (53.1) | 0.856 |
| Family history of breast cancer, n (%) | |  |  |  |
| No | 463 (84.5) | 1,008 (79.8) | 1,335 (82.4) |  |
| Yes | 85 (15.5) | 255 (20.2) | 286 (17.6) | 0.042 |
| History of personal benign breast disease, n (%) | | |  |  |
| No | 368 (67.2) | 897 (71.0) | 1,143 (70.5) |  |
| Yes | 180 (32.8) | 366 (29.0) | 478 (29.5) | 0.233 |
| Mammography before inclusion, n (%) | |  |  |  |
| No | 113 (20.6) | 264 (20.9) | 384 (23.7) |  |
| Yes | 435 (79.4) | 999 (79.1) | 1,237 (76.3) | 0.128 |
| ER status, n (%) |  |  |  |  |
| ER - | 43 (7.8) | 145 (11.5) | 352 (21.7) |  |
| ER + | 401 (73.2) | 898 (71.1) | 1,053 (65.0) |  |
| Missing | 104 (19.0) | 220 (17.4) | 216 (13.3) | <0.001 |
| PR status, n (%) |  |  |  |  |
| PR - | 121 (22.1) | 279 (22.1) | 580 (35.8) |  |
| PR + | 298 (54.4) | 731 (57.9) | 780 (48.1) |  |
| Missing | 129 (23.5) | 253 (20.0) | 261 (16.1) | <0.001 |

The analyses were done on the three main type of grade after excluding grade 4 (1 case) and those with missing grade information (971 cases)

P values estimated based on Kruskal Wallis test for continuous variables and Chi-square test for categorical variables
SD: Standard deviation, MET: Metabolic Equivalent of Task, MHT: menopausal hormone replacement therapy, Menopausal status at index date: date of diagnosis of the case in the case-control pair, ER: estrogen receptor, PR: estrogen receptor

**Supplementary** **Table S2**: Demographic and lifestyle characteristics of cases according to histological type of breast cancer at diagnosis in the case-control study nested within the E3N cohort, France, 1990-2008

| Characteristics | IDC  n (%)=2,940 (76.1%) | ILC  n (%)=682 (17.7%) | ITC  n (%)=131 (3.4%) | ID and ILC  n (%)=110 (2.8%) | *P* value |  |  |
| --- | --- | --- | --- | --- | --- | --- | --- |
| Cumulative airborne cadmium exposure (mg/m^2^), mean ± SD | 13.1 ± 68.9 | 10.3 ± 27.7 | 19.2 ± 70.9 | 16.5 ± 66.9 | 0.227 |  |  |
| Age at recruitment (years), mean ± SD | 49.6 ± 6.3 | 50.3 ± 6.4 | 49.2 ± 5.4 | 49.3 ± 6.0 | 0.078 |  |  |
| Age at diagnosis (years), mean ± SD | 59.1 ± 7.7 | 59.8 ± 7.8 | 58.9 ± 6.7 | 58.4 ± 7.5 | 0.126 |  |  |
| Alcohol drinking (g/day), n (%) | | |  |  |  |  |  |
| Never | 271 (9.2) | 59 (8.7) | 14 (10.7) | 4 (3.6) |  |  |  |
| < 6.7 | 864 (29.4) | 189 (27.7) | 34 (25.9) | 24 (21.8) |  |  |  |
| ≥ 6.7 | 1,270 (43.2) | 313 (45.9) | 64 (48.9) | 54 (49.1) |  |  |  |
| Missing | 535 (18.2) | 121 (17.7) | 19 (14.5) | 28 (25.5) | 0.137 |  |  |
| Body Mass Index (kg/m²), n (%) | | | |  |  |  |  |
| < 25 | 2,446 (83.2) | 568 (83.3) | 115 (87.8) | 88 (80.0) |  |  |  |
| 25 - <30 | 416 (14.2) | 91 (13.3) | 14 (10.7) | 19 (18.2) |  |  |  |
| ≥ 30 | 78 (2.6) | 23 (3.4) | 2 (1.5) | 2 (1.8) | 0.512 |  |  |
| Smoking status, n (%) |  |  |  |  |  |  |  |
| Never | 1,601 (54.5) | 355 (52.1) | 70 (53.4) | 52 (47.3) |  |  |  |
| Current | 413 (14.0) | 114 (16.7) | 25 (18.1) | 19 (17.3) |  |  |  |
| Former | 926 (31.5) | 213 (31.2) | 36 (27.5) | 39 (35.4) | 0.267 |  |  |
| Status of birthplace, n (%) |  |  |  |  |  |  |  |
| Rural | 778 (26.5) | 182 (26.7) | 28 (21.4) | 27 (24.6) |  |  |  |
| Urban | 1,898 (64.5) | 434 (63.6) | 93 (71.0) | 72 (65.4) |  |  |  |
| Missing | 264 (9.0) | 66 (9.7) | 10 (7.6) | 11 (10.0) | 0.803 |  |  |
| Physical activity (METs-h/week), n (%) | |  |  |  |  |  |  |
| < 25.3 | 726 (24.7) | 163 (23.9) | 32 (24.4) | 24 (21.8) |  |  |  |
| 25.3 - 37.3 | 933 (31.7) | 205 (30.1) | 40 (30.5) | 45 (40.9) |  |  |  |
| 37.4 - 56.9 | 764 (26.0) | 198 (29.0) | 38 (29.0) | 23 (20.9) |  |  |  |
| ≥ 57.0 | 517 (17.6) | 116 (17.0) | 21 (16.0) | 18 (16.4) | 0.541 |  |  |
| Education, n (%) |  |  |  |  |  |  |  |
| Secondary | 363 (12.3) | 70 (10.3) | 17 (13.0) | 14 (12.7) |  |  |  |
| 1 to 2 year university degree | 1,504 (51.2) | 344 (50.4) | 59 (45.0) | 55 (50.0) |  |  |  |
| ≥ 3 year university degree | 1,073 (36.5) | 268 (39.3) | 55 (42.0) | 41 (37.3) | 0.528 |  |  |
| Menopausal status, n (%) |  |  |  |  |  |  |  |
| Premenopausal | 598 (20.3) | 122 (17.9) | 21 (16.0) | 23 (20.9) |  |  |  |
| Postmenopausal | 2,342 (79.7) | 560 (82.1) | 110 (84.0) | 87 (79.1) | 0.338 |  |  |
| Use of oral contraceptives, n (%) | |  |  |  |  |  |  |
| No | 1,203 (40.9) | 270 (39.6) | 49 (37.4) | 45 (40.9) |  |  |  |
| Yes | 1,737 (59.1) | 412 (60.4) | 82 (62.6) | 65 (59.1) | 0.809 |  |  |
| Use of MHT, n (%) |  |  |  |  |  |  |  |
| No | 2,439 (83.0) | 557 (81.7) | 107 (81.7) | 87 (79.1) |  |  |  |
| Yes | 501 (17.0) | 125 (18.3) | 24 (18.3) | 23 (20.9) | 0.639 |  |  |
| Parity & Age at First Pregnancy (AFP), n (%) | |  |  |  |  |  |  |
| 0 | 402 (13.7) | 78 (11.4) | 16 (12.2) | 17 (15.5) |  |  |  |
| 1-2 & AFP < 30 | 1,463 (49.8) | 324 (47.5) | 69 (52.7) | 53 (48.2) |  |  |  |
| 1-2 & AFP ≥ 30 | 322 (10.9) | 99 (14.5) | 11 (8.4) | 14 (12.7) |  |  |  |
| ≥ 3 | 753 (25.6) | 181 (26.5) | 35 (26.7) | 26 (23.6) | 0.258 |  |  |
| Age at menarche, n (%) |  |  |  |  |  |  |  |
| < 12 | 624 (21.2) | 138 (20.2) | 35 (26.7) | 31 (28.2) |  |  |  |
| 12-13 | 1,552 (52.8) | 366 (53.7) | 66 (50.4) | 49 (44.5) |  |  |  |
| ≥ 14 | 764 (26.0) | 178 (26.1) | 30 (22.9) | 30 (27.3) | 0.344 |  |  |
| Breastfeeding, n (%) |  |  |  |  |  |  |  |
| No | 1,409 (47.9) | 302 (44.3) | 65 (49.6) | 57 (51.8) |  |  |  |
| Yes | 1,531 (52.1) | 380 (55.7) | 66 (50.4) | 53 (48.2) | 0.251 |  |  |
| Family history of breast cancer, n (%) | |  |  |  |  |  |  |
| No | 2,411 (82.0) | 557 (81.7) | 111 (84.7) | 82 (74.6) |  |  |  |
| Yes | 529 (18.0) | 125 (18.3) | 20 (15.3) | 28 (25.4) | 0.192 |  |  |
| History of personal benign breast disease, n (%) | | |  |  |  |  |  |
| No | 2,063 (70.2) | 482 (70.7) | 83 (63.4) | 76 (69.1) |  |  |  |
| Yes | 877 (29.8) | 200 (29.3) | 48 (36.6) | 34 (30.9) | 0.394 |  |  |
| Mammography before inclusion, n (%) | |  |  |  |  |  |  |
| No | 660 (22.4) | 148 (21.7) | 24 (18.3) | 24 (21.8) |  |  |  |
| Yes | 2,280 (77.6) | 534 (78.3) | 107 (81.7) | 86 (78.2) | 0.717 |  |  |
| ER status, n (%) |  |  |  |  |  |  |  |
| ER - | 522 (17.8) | 69 (10.1) | 10 (7.6) | 9 (9.2) |  |  |  |
| ER + | 1,921 (65.3) | 481 (70.5) | 94 (71.8) | 81 (73.6) |  |  |  |
| Missing | 497 (16.9) | 132 (19.4) | 27 (20.6) | 20 (18.2) | < 0.001 |  |  |
| PR status, n (%) |  |  |  |  |  |  |  |
| PR - | 875 (29.8) | 162 (23.7) | 36 (27.5) | 20 (18.2) |  |  |  |
| PR + | 1,488 (50.6) | 375 (55.0) | 62 (47.3) | 68 (61.8) |  |  |  |
| Missing | 577 (19.6) | 145 (21.3) | 33 (25.2) | 22 (20.0) | 0.005 |  |  |

The analyses were done on the four main type of histology after excluding other histology cases (256 cases) and those with missing histology information (288 cases)

P values estimated based on Kruskal Wallis test for continuous variables and Chi-square test for categorical variables
SD: Standard deviation, MET: Metabolic Equivalent of Task, MHT: menopausal hormone therapy, Menopausal status at index date: date of diagnosis of the case in the case-control pair, ER: estrogen receptor, PR: progesterone receptor

**Supplementary Table S3**: Distribution of baseline demographic and risk factors of breast cancer between women without and with stage information in the case-control study nested within E3N cohort, France, 1990-2008

| Characteristics | Women without stage information | Women with stage information | *P* value |
| --- | --- | --- | --- |
|  | n (%), 477 (10.8%) | n (%), 3,924 (89.2%) |  |
| Cumulative airborne cadmium exposure (mg/m^2^), mean ± SD | 14.7 ± 49.9 | 12.9 ± 63.2 | 0.346 |
| Age (years), mean (SD) | 49.5 ± 6.8 | 49.8 ± 6.3 | 0.131 |
| Age at diagnosis (years), mean ± SD | 60.8 ± 8.7 | 59.2 ± 7.7 | <0.001 |
| Alcohol drinking (g/day) |  |  |  |
| 0 | 43 (9.0) | 356 (9.1) |  |
| 0-6.7 | 129 (27.0) | 1,123 (28.6) |  |
| ≥ 6.7 | 194 (40.7) | 1,720 (43.8) |  |
| Missing | 111 (23.3) | 725 (18.5) | 0.090 |
| Body Mass Index (kg/m²) |  |  |  |
| <25 | 382 (80.1) | 3,264 (83.2) |  |
| 25-30 | 80 (16.8) | 548 (14.0) |  |
| ≥ 30 | 15 (3.1) | 112 (2.8) | 0.227 |
| Smoking status, n (%) |  |  |  |
| Never | 265 (55.6) | 2,116 (53.9) |  |
| Current | 60 (12.6) | 597 (15.2) |  |
| Former | 152 (31.9) | 1,211 (30.9) | 0.312 |
| Status of birthplace, n (%) |  |  |  |
| Rural | 133 (27.9) | 1,042 (26.6) |  |
| Urban | 296 (62.0) | 2,525 (64.3) |  |
| Missing | 48 (10.1) | 357 (9.1) | 0.589 |
| Physical activity (METs-h/week), n (%) | |  |  |
| < 25.3 | 133 (27.9) | 962 (24.5) |  |
| 25.3-37.3 | 128 (26.8) | 1,239 (31.6) |  |
| 37.4-56.9 | 134 (28.1) | 1,033 (26.3) |  |
| ≥ 57.0 | 82 (17.2) | 690 (17.6) | 0.136 |
| Education, n (%) |  |  |  |
| Secondary | 69 (14.5) | 474 (12.1) |  |
| 1- to 2-year university degree | 243 (50.9) | 1,993 (50.8) |  |
| ≥ 3 year university degree | 165 (34.6) | 1,457 (37.1) | 0.258 |
| Menopausal status, n (%) |  |  |  |
| Premenopausal | 93 (19.5) | 759 (19.3) |  |
| Postmenopausal | 384 (80.5) | 3,165 (80.7) | 0.936 |
| Use of oral contraceptives, n (%) |  |  |  |
| No | 206 (43.2) | 1,601 (40.8) |  |
| Yes | 271 (56.8) | 2,323(59.2) | 0.317 |
| Use of HRT, n (%) |  |  |  |
| No | 416 (87.2) | 3,226 (82.2) |  |
| Yes | 61 (12.8) | 698 (17.8) | 0.006 |
| Parity & Age at First Pregnancy (AFP), n (%) | |  |  |
| 0 | 60 (12.6) | 519 (13.2) |  |
| 0-2 & AFP < 30 | 225 (47.2) | 1,913 (48.8) |  |
| 0-2 & AFP ≥ 30 | 52 (10.9) | 448 (11.4) |  |
| ≥ 3 | 135 (28.3) | 1,014 (25.8) |  |
| Missing | 5 (1.0) | 30 (0.8) | 0.759 |
| Age at menarche, n (%) |  |  |  |
| < 12 | 114 (23.9) | 841 (21.4) |  |
| 12-14 | 237 (49.7) | 2,063 (52.6) |  |
| ≥ 14 | 126 (26.4) | 1,020 (26.0) | 0.387 |
| Breastfeeding, n (%) |  |  |  |
| No | 238 (49.9) | 1,861 (47.4) |  |
| Yes | 239 (50.1) | 2,063 (52.6) | 0.308 |
| Family history of breast cancer, n (%) | |  |  |
| No | 402 (84.3) | 3,200 (81.6) |  |
| Yes | 75 (15.7) | 724 (18.4) | 0.145 |
| History of personal benign breast disease, n (%) | |  |  |
| No | 344 (72.1) | 2,752 (70.1) |  |
| Yes | 133 (27.9) | 1,172 (29.9) | 0.370 |
| Mammography before inclusion, n (%) | |  |  |
| No | 134 (28.1) | 856 (21.8) |  |
| Yes | 343 (71.9) | 3,068 (78.2) | 0.002 |

SD: Standard deviation, MET: Metabolic Equivalent of Task, HRT: menopausal hormone replacement therapy, CIE: cumulative index of exposure to cadmium. Menopausal status at index date: date of diagnosis of the case in the case-control pair

**Supplementary Table S4**: Distribution of baseline demographic and risk factors of breast cancer between women without and with grade information in the case-control study nested within E3N cohort, France, 1990-2008

| Characteristics | Women without grade information | Women with grade information | *P* value |
| --- | --- | --- | --- |
|  | n (%), 968 (22.0%) | n (%), 3,433 (78.0%) |  |
| Cumulative airborne cadmium exposure (mg/m^2^), mean ± SD | 14.4 ± 50.3 | 12.7 ± 64.9 | 0.449 |
| Age (years), mean (SD) | 49.8 ± 6.6 | 49.8 ± 6.3 | 0.752 |
| Age at diagnosis (years), mean ± SD | 59.0 ± 8.4 | 59.5 ± 7.6 | 0.037 |
| Alcohol drinking (g/day) |  |  |  |
| 0 | 88 (9.1) | 311(9.1) |  |
| 0-6.7 | 269 (27.8) | 983 (28.6) |  |
| ≥ 6.7 | 400 (41.3) | 1,514 (44.1) |  |
| Missing | 211 (21.8) | 625 (18.2) | 0.084 |
| Body Mass Index (kg/m²) |  |  |  |
| <25 | 801 (82.7) | 2,845 (82.9) |  |
| 25-30 | 136 (14.1) | 492 (14.3) |  |
| ≥ 30 | 31 (3.2) | 96 (2.8) | 0.789 |
| Smoking status, n (%) |  |  |  |
| Never | 514 (53.1) | 1,867 (54.4) |  |
| Current | 151 (15.6) | 506 (14.7) |  |
| Former | 303 (31.3) | 1,060 (30.9) | 0.723 |
| Status of birthplace, n (%) |  |  |  |
| Rural | 249 (25.7) | 926 (27.0) |  |
| Urban | 614 (63.4) | 2,207 (64.3) |  |
| Missing | 105 (10.9) | 300 (8.7) | 0.124 |
| Physical activity (METs-h/week), n (%) | |  |  |
| < 25.3 | 250 (25.8) | 845 (24.6) |  |
| 25.3-37.3 | 298 (30.8) | 1,069 (31.1) |  |
| 37.4-56.9 | 264 (27.3) | 903 (26.3) |  |
| ≥ 57.0 | 156 (16.1) | 616 (17.9) | 0.535 |
| Education, n (%) |  |  |  |
| Secondary | 121 (12.5) | 422 (12.3) |  |
| 1- to 2-year university degree | 496 (51.2) | 1,740 (50.7) |  |
| ≥ 3 year university degree | 351 (36.3) | 1,271 (37.0) | 0.909 |
| Menopausal status, n (%) |  |  |  |
| Premenopausal | 227 (23.5) | 625 (18.2) |  |
| Postmenopausal | 741 (76.5) | 2,808 (81.8) | <0.001 |
| Use of oral contraceptives, n (%) |  |  |  |
| No | 409 (42.3) | 1,398 (40.7) |  |
| Yes | 559 (57.7) | 2,035 (59.3) | 0.393 |
| Use of HRT, n (%) |  |  |  |
| No | 821 (84.8) | 2,821 (82.2) |  |
| Yes | 147 (15.2) | 612 (17.8) | 0.055 |
| Parity & Age at First Pregnancy (AFP), n (%) | |  |  |
| 0 | 132 (13.6) | 447 (13.0) |  |
| 0-2 & AFP < 30 | 453 (46.8) | 1,685 (49.1) |  |
| 0-2 & AFP ≥ 30 | 117 (12.1) | 383 (11.1) |  |
| ≥ 3 | 261 (27.0) | 888 (25.9) |  |
| Missing | 5 (0.5) | 30 (0.9) | 0.531 |
| Age at menarche, n (%) |  |  |  |
| < 12 | 218 (22.5) | 737 (21.5) |  |
| 12-14 | 495 (51.1) | 1,805 (52.6) |  |
| ≥ 14 | 255 (26.4) | 891 (25.9) | 0.694 |
| Breastfeeding, n (%) |  |  |  |
| No | 473 (48.9) | 1,626 (47.4) |  |
| Yes | 495 (51.1) | 1,807 (52.6) | 0.409 |
| Family history of breast cancer, n (%) | |  |  |
| No | 795 (82.1) | 2,807 (81.8) |  |
| Yes | 173 (17.9) | 626 (18.2) | 0.796 |
| History of personal benign breast disease, n (%) | |  |  |
| No | 687 (71.0) | 2,409 (70.2) |  |
| Yes | 281 (29.0) | 1,024 (29.8) | 0.631 |
| Mammography before inclusion, n (%) | |  |  |
| No | 229 (23.7) | 761 (22.2) |  |
| Yes | 739 (76.3) | 2,672 (77.8) | 0.327 |

SD: Standard deviation, MET: Metabolic Equivalent of Task, HRT: menopausal hormone replacement therapy, CIE: cumulative index of exposure to cadmium. Menopausal status at index date: date of diagnosis of the case in the case-control pair

**Supplementary Table S5**: Distribution of baseline demographic and risk factors of breast cancer between women without and with histology information in the case-control study nested within E3N cohort, France, 1990-2008

| Characteristics | Women without histology information | Women with histology information | *P* value |
| --- | --- | --- | --- |
|  | n (%), 281 (6.4%) | n (%), 4,120 (93.6%) |  |
| Cumulative airborne cadmium exposure (mg/m^2^), mean ± SD | 16.0 ± 54.2 | 12.9 ± 62.5 | 0.152 |
| Age (years), mean (SD) | 50.5 ± 7.1 | 49.7 ± 6.3 | 0.162 |
| Age at diagnosis (years), mean ± SD | 64.1 ± 7.6 | 59.1 ± 7.7 | <0.001 |
| Alcohol drinking (g/day) |  |  |  |
| 0 | 27 (9.6) | 372 (9.0) |  |
| 0-6.7 | 65 (23.1) | 1,187 (28.8) |  |
| ≥ 6.7 | 115 (40.9) | 1,799 (43.7) |  |
| Missing | 74 (26.3) | 762 (18.5) | 0.007 |
| Body Mass Index (kg/m²) |  |  |  |
| <25 | 226 (80.4) | 3,420 (83.0) |  |
| 25-30 | 44 (15.7) | 584 (14.2) |  |
| ≥ 30 | 11 (3.9) | 116 (2.8) | 0.423 |
| Smoking status, n (%) |  |  |  |
| Never | 158 (56.2) | 2,223 (54.0) |  |
| Current | 43 (15.3) | 614 (14.9) |  |
| Former | 80 (28.5) | 1,283 (31.1) | 0.642 |
| Status of birthplace, n (%) |  |  |  |
| Rural | 76 (27.1) | 1,099 (26.7) |  |
| Urban | 174 (61.9) | 2,647 (64.2) |  |
| Missing | 31 (11.0) | 374 (9.1) | 0.515 |
| Physical activity (METs-h/week), n (%) | |  |  |
| < 25.3 | 83 (29.5) | 1,012 (24.6) |  |
| 25.3-37.3 | 66 (23.5) | 1,301 (31.6) |  |
| 37.4-56.9 | 82 (29.2) | 1,085 (26.3) |  |
| ≥ 57.0 | 50 (17.8) | 722 (17.5) | 0.030 |
| Education, n (%) |  |  |  |
| Secondary | 47 (16.7) | 496 (12.0) |  |
| 1- to 2-year university degree | 141 (50.2) | 2,095 (50.9) |  |
| ≥ 3 year university degree | 93 (33.1) | 1,529 (37.1) | 0.053 |
| Menopausal status, n (%) |  |  |  |
| Premenopausal | 18 (6.4) | 834 (20.2) |  |
| Postmenopausal | 263 (93.6) | 3,286 (79.8) | <0.001 |
| Use of oral contraceptives, n (%) |  |  |  |
| No | 132 (47.0) | 1,675 (40.7) |  |
| Yes | 149 (53.0) | 2,445 (59.3) | 0.037 |
| Use of HRT, n (%) |  |  |  |
| No | 241 (85.8) | 3,401 (82.6) |  |
| Yes | 40 (14.2) | 719 (17.4) | 0.167 |
| Parity & Age at First Pregnancy (AFP), n (%) | |  |  |
| 0 | 34 (12.1) | 545 (13.2) |  |
| 0-2 & AFP < 30 | 123 (43.8) | 2,015 (48.9) |  |
| 0-2 & AFP ≥ 30 | 30 (10.7) | 470 (11.4) |  |
| ≥ 3 | 90 (32.0) | 1,059 (25.7) |  |
| Missing | 4 (1.4) | 31 (0.8) | 0.120 |
| Age at menarche, n (%) |  |  |  |
| < 12 | 60 (21.3) | 895 (21.7) |  |
| 12-14 | 139 (49.5) | 2,161 (52.5) |  |
| ≥ 14 | 82 (29.2) | 1,064 (25.8) | 0.449 |
| Breastfeeding, n (%) |  |  |  |
| No | 136 (48.4) | 1,963 (47.6) |  |
| Yes | 145 (51.6) | 2,157 (52.4) | 0.807 |
| Family history of breast cancer, n (%) | |  |  |
| No | 234 (83.3) | 3,368 (81.7) |  |
| Yes | 47 (16.7) | 752 (18.3) | 0.521 |
| History of personal benign breast disease, n (%) | |  |  |
| No | 208 (74.0) | 2,888 (70.1) |  |
| Yes | 73 (26.0) | 1,232 (29.9) | 0.163 |
| Mammography before inclusion, n (%) | |  |  |
| No | 77 (27.4) | 913 (22.2) |  |
| Yes | 204 (72.6) | 3,207 (77.8) | 0.042 |

SD: Standard deviation, MET: Metabolic Equivalent of Task, HRT: menopausal hormone replacement therapy, CIE: cumulative index of exposure to cadmium. Menopausal status at index date: date of diagnosis of the case in the case-control pair

**Supplementary** **Table S6:** Odds ratio and 95% confidence intervals (OR, 95% CI) for the association between mean airborne cadmium exposure and risk of breast cancer according to stage in pre- and post-menopausal women at index date: The case-control study nested within the E3N cohort, France, 1990-2008.

|  | **Stage I** | |  | **Stage II** | |  | **Stages III-IV** | |
| --- | --- | --- | --- | --- | --- | --- | --- | --- |
| Cumulative airborne cadmium exposure (mg/m^2^) | n cases/ controls | OR (95% CI) |  | n cases/ controls | OR (95% CI) |  | n cases/ controls | OR(95% CI) |
| Premenopausal |  |  |  |  |  |  |  |  |
| < 0.0017 | 111/102 | Ref |  | 78/70 | Ref |  | 29/29 | Ref |
| > 0.0017 - 0.0188 | 103/101 | 1.04 (0.60-1.80) |  | 61/52 | 0.91 (0.45-1.87) |  | 29/19 | 1.35 (0.34-5.45) |
| > 0.0188 - 0.0779 | 102/84 | 1.23 (0.69-2.18) |  | 56/49 | 1.48 (0.69-3.18) |  | 19/15 | 0.80 (0.19-3.47) |
| > 0.0779 - 0.288 | 57/54 | 1.10 (0.54-2.24) |  | 41/42 | 0.63 (0.28-1.42) |  | 15/17 | 0.98 (0.15-6.26) |
| > 0.288 | 54/59 | 1.08 (0.55-2.13) |  | 26/36 | 0.47 (0.18-1.25) |  | 8/14 | 0.26 (0.03-2.61) |
| *P* trend |  | 0.733 |  |  | 0.195 |  |  | 0.401 |
| *P* likelihood* |  | 0.965 |  |  | 0.171 |  |  | 0.737 |
| *P* heterogeneity** |  |  |  |  |  |  |  | 0.685 |
| Postmenopausal |  |  |  |  |  |  |  |  |
| < 0.0017 | 375/375 | Ref |  | 181/174 | Ref |  | 44/63 | Ref |
| > 0.0017 - 0.0188 | 357/384 | 0.91 (0.72-1.14) |  | 176/219 | 0.76 (0.54-1.06) |  | 52/45 | 1.69 (0.87-3.28) |
| > 0.0188 - 0.0779 | 395/428 | 0.82 (0.65-1.03) |  | 199/193 | 1.03 (0.73-1.44) |  | 53/38 | 2.17 (1.06-4.46) |
| > 0.0779 - 0.288 | 434/421 | 0.96 (0.76-1.21) |  | 194/199 | 0.90 (0.63-1.28) |  | 38/62 | 0.81 (0.38-1.71) |
| > 0.288 | 447/427 | 0.95 (0.75-1.21) |  | 233/211 | 1.11 (0.77-1.57) |  | 56/41 | 2.18 (1.03-4.60) |
| *P* trend |  | 0.890 |  |  | 0.309 |  |  | 0.232 |
| *P* likelihood* |  | 0.467 |  |  | 0.184 |  |  | 0.007 |
| *P* heterogeneity** |  |  |  |  |  |  |  | 0.116 |

Multivariable models were adjusted for physical activity, smoking status, level of education, body mass index (BMI), previous family history

of breast cancer, personal history of breast cancer, age at menarche, age at first full-term pregnancy, parity, breastfeeding, oral contraceptive

use, menopausal hormone replacement therapy use (HRT) and status of birthplace

Menopausal status at index date: date of diagnosis of the case in the case-control pair

P likelihood*: P-values from likelihood ratio test comparing the statistically significance of the global effect of the quintiles

P heterogeneity**: comparing heterogeneity of associations across breast cancer stage at diagnosis

**Supplementary** **Table S7:** Sensitivity analyses of the multivariable model additionally adjusted for mammographic examination before inclusion: Odds ratio and 95% confidence intervals (OR, 95% CI) for the association of quintiles of the cumulative airborne cadmium exposure with risk of breast cancer according to breast cancer stage in the case-control study nested within the E3N cohort, France, 1990-2008.

| Cumulative airborne cadmium exposure (mg/m^2^) | n cases/ controls | OR (95% CI) ^a^ | *P* trend | *P* likelihood | | *P* heterogeneity | | |
| --- | --- | --- | --- | --- | --- | --- | --- | --- |
| Stage I |  |  |  |  | | |  | |
| ≤ 0.011 | 468/484 | Ref |  |  | | |  | |
| > 0.011 - 0.105 | 502/453 | 1.1 (0.9-1.3) |  |  | | |  | |
| > 0.105 - 0.375 | 477/487 | 1.2 (1.0-1.4) |  |  | | |  | |
| > 0.375 - 1.32 | 448/478 | 1.0 (0.9-1.3) |  |  |  | | |  |
| > 1.32 | 475/468 | 1.0 (0.8-1.2) | 0.835 | 0.502 | | |  | |
| Stage II |  |  |  |  | | |  | |
| ≤ 0.011 | 244/240 | Ref |  |  | | |  | |
| > 0.011 - 0.105 | 225/266 | 0.8 (0.6-1.1) |  |  | | |  | |
| > 0.105 - 0.375 | 264/240 | 1.1 (0.8-1.4) |  |  | | |  | |
| > 0.375 - 1.32 | 236/244 | 1.0 (0.7-1.3) |  |  | | |  | |
| > 1.32 | 247/226 | 1.1 (0.8-1.5) | 0.297 | 0.208 | | |  | |
| Stages III-IV |  |  |  |  | | |  | |
| ≤ 0.011 | 76/65 | Ref |  |  | | |  | |
| > 0.011 - 0.105 | 70/77 | 0.8 (0.5-1.4) |  |  | | |  | |
| > 0.105 - 0.375 | 73/69 | 0.6 (0.4-1.0) |  |  | | |  | |
| > 0.375 - 1.32 | 54/59 | 0.8 (0.4-1.4) |  |  | | |  | |
| > 1.32 | 65/68 | 0.7 (0.4-1.3) | 0.244 | 0.470 | | | 0.455 | |

^a^ Multivariable models were adjusted for physical activity, smoking status, level of education, body mass index (BMI), age at menarche, age at first full-term pregnancy (AFP), parity, breastfeeding, oral contraceptive use, menopausal hormone replacement therapy use (HRT), status of birthplace, previous family history of breast cancer (FHBC), personal history of benign breast disease, and mammographic examination before inclusion

P likelihood: P-values from likelihood ratio test comparing the statistically significance of the global effect of the quintiles

P heterogeneity: comparing heterogeneity of associations across breast cancer stage at diagnosis

**Supplementary** **Table S8:** Odds ratio and 95% confidence intervals (OR, 95% CI) for the association of quintiles of the mean airborne cadmium exposure with risk of breast cancer according to the stage in the case-control study nested within the E3N cohort, France, 1990-2008.

| Cumulative airborne cadmium exposure (mg/m^2^) | n cases/ controls | OR (95% CI) ^a^ | *P* trend | *P* likelihood | | *P* heterogeneity | | |
| --- | --- | --- | --- | --- | --- | --- | --- | --- |
| Stage I |  |  |  |  | | |  | |
| ≤ 0.011 | 468/484 | Ref |  |  | | |  | |
| > 0.011 - 0.105 | 502/453 | 1.2 (0.9-1.4) |  |  | | |  | |
| > 0.105 - 0.375 | 477/487 | 1.0 (0.9-1.2) |  |  | | |  | |
| > 0.375 - 1.32 | 448/478 | 1.0 (0.8-1.2) |  |  |  | | |  |
| > 1.32 | 475/468 | 1.0 (0.9-1.3) | 0.743 | 0.473 | | |  | |
| Stage II |  |  |  |  | | |  | |
| ≤ 0.011 | 244/240 | Ref |  |  | | |  | |
| > 0.011 - 0.105 | 225/266 | 0.8 (0.7-1.1) |  |  | | |  | |
| > 0.105 - 0.375 | 264/240 | 1.2 (0.9-1.6) |  |  | | |  | |
| > 0.375 - 1.32 | 236/244 | 1.0 (0.8-1.4) |  |  | | |  | |
| > 1.32 | 247/226 | 1.1 (0.8-1.5) | 0.234 | 0.157 | | |  | |
| Stages III-IV |  |  |  |  | | |  | |
| ≤ 0.011 | 76/65 | Ref |  |  | | |  | |
| > 0.011 - 0.105 | 70/77 | 0.8 (0.5-1.3) |  |  | | |  | |
| > 0.105 - 0.375 | 73/69 | 0.9 (0.5-1.5) |  |  | | |  | |
| > 0.375 - 1.32 | 54/59 | 0.7 (0.4-1.2) |  |  | | |  | |
| > 1.32 | 65/68 | 0.7 (0.4-1.3) | 0.247 | 0.698 | | | 0.333 | |

^a^ Multivariable models were adjusted for physical activity, smoking status, level of education, body mass index (BMI), age at menarche, age at first full-term pregnancy (AFP), parity, breastfeeding, oral contraceptive use, menopausal hormone replacement therapy use (HRT), status of birthplace, previous family history of breast cancer (FHBC), and personal history of benign breast disease

P likelihood: P-values from likelihood ratio test comparing the statistically significance of the global effect of the quintiles

P heterogeneity: comparing heterogeneity of associations across breast cancer stage at diagnosis

**Supplementary** **Fig.S1: Cubic spline modelling of the relationship between cumulative airborne cadmium exposure and risk of invasive tubular breast cancer** i**n the case-control study nested within the E3N cohort, France, 1990-2008.** Multivariable adjusted OR (continuous line) and 95% CI (dotted line) obtained using four-knot restricted cubic splines with the minimum value used as reference. Models were adjusted for physical activity, smoking status, alcohol intake, level of education, body mass index, previous family history of breast cancer, personal history of benign breast disease, age at menarche, age at first full-term pregnancy, parity, breastfeeding, oral contraceptive use, menopausal hormone therapy use and status of birthplace. (n cases/controls=131/131).

**Supplementary** **Fig.S2: Cubic spline modelling of the relationship between cumulative airborne cadmium exposure and risk of invasive ductal breast cancer** i**n the case-control study nested within the E3N cohort, France, 1990-2008.** Multivariable adjusted OR (continuous line) and 95% CI (dotted line) obtained using four-knot restricted cubic splines with the minimum value used as reference. Models were adjusted for physical activity, smoking status, alcohol intake, level of education, body mass index, previous family history of breast cancer, personal history of benign breast disease, age at menarche, age at first full-term pregnancy, parity, breastfeeding, oral contraceptive use, menopausal hormone therapy use and status of birthplace. (n cases/controls=2,940/2,940).

**Supplementary** **Fig.S3: Cubic spline modelling of the relationship between cumulative airborne cadmium exposure and risk of invasive lobular breast cancer** i**n the case-control study nested within the E3N cohort, France, 1990-2008.** Multivariable adjusted OR (continuous line) and 95% CI (dotted line) obtained using four-knot restricted cubic splines with the minimum value used as reference. Models were adjusted for physical activity, smoking status, alcohol intake, level of education, body mass index, previous family history of breast cancer, personal history of benign breast disease, age at menarche, age at first full-term pregnancy, parity, breastfeeding, oral contraceptive use, menopausal hormone therapy use and status of birthplace. (n cases/controls=682/682).

**Supplementary** **Fig.S4: Cubic spline modelling of the relationship between cumulative airborne cadmium exposure and risk of invasive mixt (ductal lobular) breast cancer** i**n the case-control study nested within the E3N cohort, France, 1990-2008.** Multivariable adjusted OR (continuous line) and 95% CI (dotted line) obtained using four-knot restricted cubic splines with the minimum value used as reference. Models were adjusted for physical activity, smoking status, alcohol intake, level of education, body mass index, previous family history of breast cancer, personal history of benign breast disease, age at menarche, age at first full-term pregnancy, parity, breastfeeding, oral contraceptive use, menopausal hormone therapy use and status of birthplace. (n cases/controls=110/110).

**Supplementary** **Fig.S5: Cubic spline modelling of the relationship between cumulative airborne cadmium exposure and risk of stage I breast cancer** i**n the case-control study nested within the E3N cohort, France, 1990-2008.** Multivariable adjusted OR (continuous line) and 95% CI (dotted line) obtained using four-knot restricted cubic splines with the minimum value used as reference. Models were adjusted for physical activity, smoking status, alcohol intake, level of education, body mass index, previous family history of breast cancer, personal history of benign breast disease, age at menarche, age at first full-term pregnancy, parity, breastfeeding, oral contraceptive use, menopausal hormone therapy use and status of birthplace. (n cases/controls=2,370/2,370).

**Supplementary** **Fig.S6: Cubic spline modelling of the relationship between cumulative airborne cadmium exposure and risk of stage II breast cancer** i**n the case-control study nested within the E3N cohort, France, 1990-2008.** Multivariable adjusted OR (continuous line) and 95% CI (dotted line) obtained using four-knot restricted cubic splines with the minimum value used as reference. Models were adjusted for physical activity, smoking status, alcohol intake, level of education, body mass index, previous family history of breast cancer, personal history of benign breast disease, age at menarche, age at first full-term pregnancy, parity, breastfeeding, oral contraceptive use, menopausal hormone therapy use and status of birthplace. (n cases/controls=1,216/1,216).

**Supplementary** **Fig.S7: Cubic spline modelling of the relationship between cumulative airborne cadmium exposure and risk of stage III-IV breast cancer** i**n the case-control study nested within the E3N cohort, France, 1990-2008.** Multivariable adjusted OR (continuous line) and 95% CI (dotted line) obtained using four-knot restricted cubic splines with the minimum value used as reference. Models were adjusted for physical activity, smoking status, alcohol intake, level of education, body mass index, previous family history of breast cancer, personal history of benign breast disease, age at menarche, age at first full-term pregnancy, parity, breastfeeding, oral contraceptive use, menopausal hormone therapy use and status of birthplace. (n cases/controls=338/338).

**Supplementary** **Fig.S7: Cubic spline modelling of the relationship between cumulative airborne cadmium exposure and risk of grade 1 breast cancer** i**n the case-control study nested within the E3N cohort, France, 1990-2008.** Multivariable adjusted OR (continuous line) and 95% CI (dotted line) obtained using four-knot restricted cubic splines with the minimum value used as reference. Models were adjusted for physical activity, smoking status, alcohol intake, level of education, body mass index, previous family history of breast cancer, personal history of benign breast disease, age at menarche, age at first full-term pregnancy, parity, breastfeeding, oral contraceptive use, menopausal hormone therapy use and status of birthplace. (n cases/controls=548/548).

**Supplementary** **Fig.S8: Cubic spline modelling of the relationship between cumulative airborne cadmium exposure and risk of grade 2 breast cancer** i**n the case-control study nested within the E3N cohort, France, 1990-2008.** Multivariable adjusted OR (continuous line) and 95% CI (dotted line) obtained using four-knot restricted cubic splines with the minimum value used as reference. Models were adjusted for physical activity, smoking status, alcohol intake, level of education, body mass index, previous family history of breast cancer, personal history of benign breast disease, age at menarche, age at first full-term pregnancy, parity, breastfeeding, oral contraceptive use, menopausal hormone therapy use and status of birthplace. (n cases/controls=1,263 /1,263).

**Supplementary** **Fig.S9: Cubic spline modelling of the relationship between cumulative airborne cadmium exposure and risk of grade 3 breast cancer** i**n the case-control study nested within the E3N cohort, France, 1990-2008.** Multivariable adjusted OR (continuous line) and 95% CI (dotted line) obtained using four-knot restricted cubic splines with the minimum value used as reference. Models were adjusted for physical activity, smoking status, alcohol intake, level of education, body mass index, previous family history of breast cancer, personal history of benign breast disease, age at menarche, age at first full-term pregnancy, parity, breastfeeding, oral contraceptive use, menopausal hormone therapy use and status of birthplace. (n cases/controls=1,621/1,621).
